# Dramatically enhanced non-Ohmic properties and maximum stored energy density in ceramic-metal nanocomposites: CaCu_3_Ti_4_O_12_/Au nanoparticles

**DOI:** 10.1186/1556-276X-8-494

**Published:** 2013-11-21

**Authors:** Wattana Tuichai, Saowalak Somjid, Bundit Putasaeng, Teerapon Yamwong, Apiwat Chompoosor, Prasit Thongbai, Vittaya Amornkitbamrung, Santi Maensiri

**Affiliations:** 1 Materials Science and Nanotechnology Program, Faculty of Science, Khon Kaen University, Khon Kaen 40002, Thailand; 2 National Metal and Materials Technology Center (MTEC), Thailand Science Park, Pathumthani 12120, Thailand; 3 Department of Physics, Faculty of Science, Khon Kaen University, Khon Kaen 40002, Thailand; 4 Nanotec-KKU Center of Excellence on Advanced Nanomaterials for Energy Production and Storage, Khon Kaen 40002, Thailand; 5 School of Physics, Institute of Science, Suranaree University, Nakhon Ratchasima 30000, Thailand

**Keywords:** Nanocomposite, Dielectric permittivity, Percolation threshold, Varistor

## Abstract

Non-Ohmic and dielectric properties of a novel CaCu_3_Ti_4_O_12_/Au nanocomposite were investigated. Introduction of 2.5 vol.% Au nanoparticles in CaCu_3_Ti_4_O_12_ ceramics significantly reduced the loss tangent while its dielectric permittivity remained unchanged. The non-Ohmic properties of CaCu_3_Ti_4_O_12_/Au (2.5 vol.%) were dramatically improved. A nonlinear coefficient of ≈ 17.7 and breakdown electric field strength of 1.25 × 10^4^ V/m were observed. The maximum stored energy density was found to be 25.8 kJ/m^3^, which is higher than that of pure CaCu_3_Ti_4_O_12_ by a factor of 8. Au addition at higher concentrations resulted in degradation of dielectric and non-Ohmic properties, which is described well by percolation theory.

## Background

Ceramic materials with high dielectric permittivity (ϵ′) have been intensively studied because of their potential for multilayer ceramic capacitor applications. The dielectric materials used in these devices must exhibit a high ϵ′ with very low loss tangent (tanδ). They also need to have a high breakdown voltage to support high-energy density storage applications. The energy density (*U*) performance of capacitors can be expressed as $U=\epsilon^{\prime }\epsilon_{0}E_{b}^{2}/2$, where *E*_b_ is electric field breakdown strength [1]. Recently, dielectric ceramics homogeneously filled with metallic particles have been of considerable scientific and technological interest. This is due to their greatly enhanced dielectric response as well as an improved tunability of ϵ′ [2-11]. Generally, ϵ′ increases rapidly in the region of the percolation threshold (PT) [4,9]. For the Ag-Ba_0.75_Sr_0.25_TiO_3_ composite [9], the large increase in ϵ′ was suggested to result from the percolation effect. Improved tunability of Ba_0.75_Sr_0.25_TiO_3_ ceramics was hypothesized to be the effect of either large induced internal electric fields within the thin Ba_0.75_Sr_0.25_TiO_3_ layer sandwiched by electrode-like metallic Ag particles or improved densification of ceramic composites. However, *E*_b_ of a metal-ceramic composite abruptly decreased as the metallic filler concentration increased to PT [4].

CaCu_3_Ti_4_O_12_ (CCTO) is one of the most interesting ceramics because it has high ϵ′ values. CCTO polycrystalline ceramics can also exhibit non-Ohmic properties [12-20]. These two properties give CCTO potential for applications in capacitor and varistor devices, respectively. Unfortunately, high tanδ (>0.05) of CCTO ceramics is still one of the most serious problems preventing its use in applications [10,12,17]. The application of CCTO ceramics in varistor devices was limited by their low nonlinear coefficient (α) and *E*_b_ values. For energy storage devices, both ϵ′ and *E*_b_ need to be enhanced in order to make high performance energy-density capacitors. Therefore, investigations to systematically improve CCTO ceramics properties are very important.

## Methods

In this work, CaCu_3_Ti_4_O_12_ powder was prepared by a solid state reaction method. First, CaCO_3_, CuO, and TiO_2_ were mixed homogeneously in ethanol for 24 h using ZrO_2_ balls. Second, the resulting mixture was dried and then ground into fine powders. Then, dried powder samples were calcined at 900°C for 6 h. HAuCl_4_, sodium citrate, and deionized water were used to prepare Au NPs by the Turkevich method [21]. CCTO/Au nanocomposites with different Au volume fractions of 0, 0.025, 0.05, 0.1, and 0.2 (abbreviated as CCTO, CCTO/Au1, CCTO/Au2, CCTO/Au3, and CCTO/Au4 samples, respectively) were prepared. CCTO and Au NPs were mixed and pressed into pellets. Finally, the pellets were sintered in air at 1,060°C for 3 h.

X-ray diffraction (XRD; Philips PW3040, Philips, Eindhoven, The Netherlands) was used to characterize the phase formation of sintered CCTO/Au nanocomposites. Scanning electron microscopy (SEM; LEO 1450VP, LEO Electron Microscopy Ltd, Cambridge, UK) coupled with energy-dispersive X-ray spectrometry (EDS) were used to characterize the microstructure of these materials. Transmission electron microscopy (TEM) (FEI Tecnai G^2^, FEI, Hillsboro, OR, USA) was used to reveal Au NPs. The polished surfaces of sintered CCTO/Au samples were coated with Au sputtered electrode. Dielectric properties were measured using an Agilent 4294A Precision Impedance Analyzer (Agilent Technologies, Santa Clara, CA, USA) over the frequency range from 10^2^ to 10^7^ Hz with an oscillation voltage of 0.5 V.

## Results and discussion

**Figure 1:**
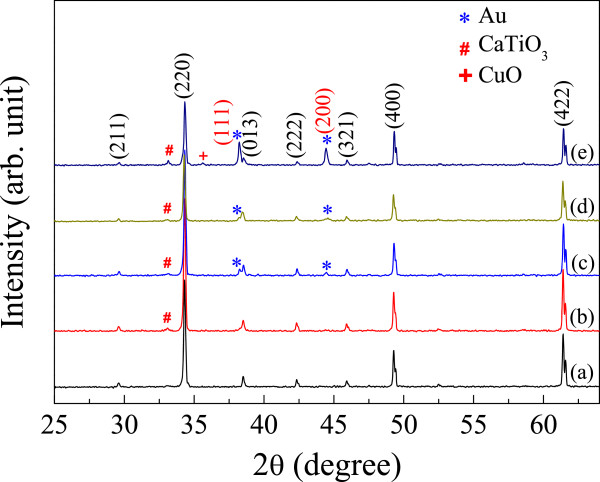
XRD patterns of (a) CCTO, (b) CCTO/Au1, (c) CCTO/Au2, (d) CCTO/Au3, and (e) CCTO/Au4 samples.

Figure 1 shows the XRD patterns of the CCTO/Au nanocomposites, confirming the major CCTO matrix phase (JCPDS 75–2188) and the minor phase of Au filler (JCPDS 04–0784). An impurity phase of CaTiO_3_ (CTO) was also observed in the XRD patterns of the CCTO/Au samples. The intensity ratio between the (111) peak of Au and the (200) peak of CCTO was found to increase with increasing Au concentrations. Lattice parameters of the CCTO phase for the CCTO, CCTO/Au1, CCTO/Au2, CCTO/Au3, and CCTO/Au4 samples were calculated to be 7.391, 7.391, 7.391, 7.390, and 7.390 Å, respectively. These parameters are nearly the same in value and are comparable to those reported in the literature [12,16,17]. This means that Au was not substituted into any sites in the CCTO lattice.

**Figure 2:**
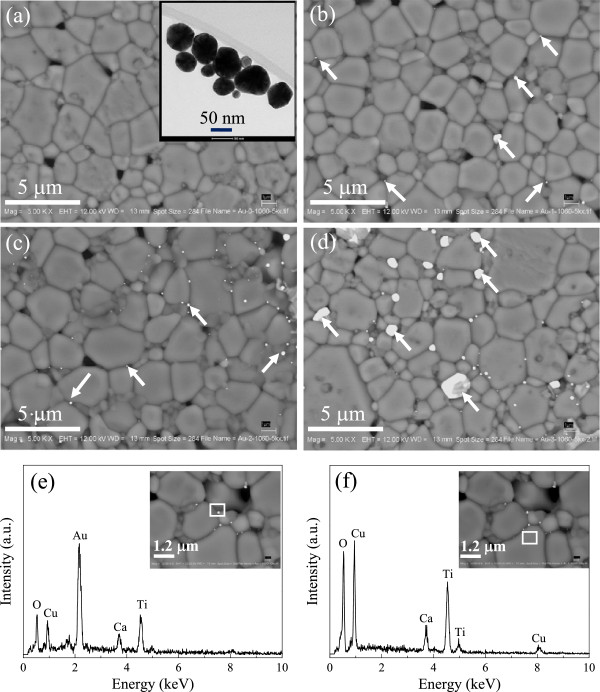
**SEM backscattered images of (a) CCTO, (b) CCTO/Au1, (c) CCTO/Au2, and (d) CCTO/Au3 samples; (e, f) EDS spectra of the CCTO/Au1 sample.** The inset of **(a)** shows TEM image of Au NPs. **(e, f)** EDS spectra of the CCTO/Au1 sample detected at a bright particle on GB and a regular grain, respectively; insets of **(e)** and **(f)** show the testing EDS points, indicated by rectangular areas.

The distribution of the Au filler in the microstructure of CCTO matrix is revealed in Figure 2a,b,c,d. The inset of Figure 2a shows the TEM image of Au NPs with particle sizes of about 50 to 100 nm. Two distinct phases were observed, consisting of regular grains and light particles appearing as spots, which are indicated by arrows. The amount and particle size of the lighter phase increased with increasing Au NP concentrations. Figure 2e,f shows the EDS spectra of the CCTO/Au1 sample at the location of a light particle (inset of panel e) and a regular grain (inset of panel f), respectively. It is important to mention that before the SEM and EDS techniques were performed, surfaces of all the CCTO/Au samples were not coated with Au sputtered layer in order to identify the Au NPs in the CCTO matrix. Therefore, the light particles are clearly indicated as Au phase. Most of Au particles are located at the grain boundary (GB) or at the triple point junction between grains.

**Figure 3:**
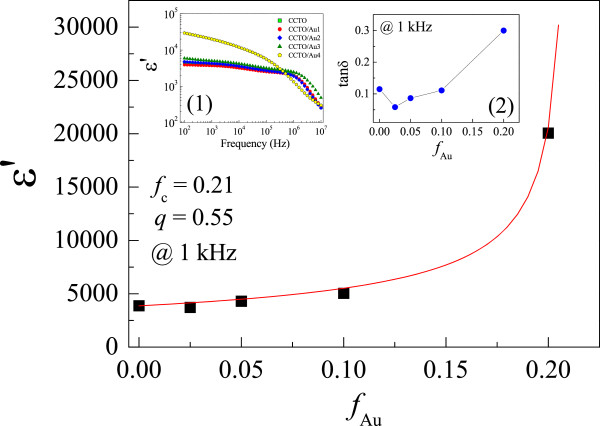
**The dependence of Au volume fraction on ϵ′ at RT for CCTO/Au nanocomposites.** The symbols and solid curve represent the experimental data and the fitted curve, respectively. Insets 1 and 2 show the frequency dependence of ϵ′ at RT and tanδ (at 1 kHz and RT) of CCTO/Au nanocomposites.

In Figure 3, ϵ′ values at 1 kHz and RT for the CCTO, CCTO/Au1, CCTO/Au2, CCTO/Au3, and CCTO/Au4 samples were found to be 3,864, 3,720, 4,293, 5,039, and 20,060, respectively. Their tanδ values were 0.115, 0.058, 0.087, 0.111, and 0.300, respectively (inset (2)). The low-frequency ϵ′ and tanδ of the CCTO, CCTO/Au1, CCTO/Au2, and CCTO/Au3 samples were slightly different (inset (1)). Both ϵ′ and tanδ were strongly enhanced as the concentration of Au NP filler was increased to 20 vol.%. Generally, dramatic changes in metal-insulator matrix composites in the critical region are attributed to the percolation effect [4,7,9,17,22-24]. A rapid increase in effective dielectric constant ($\epsilon_{\mathrm{eff}}^{\prime }$) of the composites can be described by the power law [4,9,22,24]:

(1) $$\epsilon_{\mathrm{eff}}^{\prime }=\epsilon_{\mathrm{matrix}}^{\prime }{\left|\frac{f_{c}-f}{f_{c}}\right|}^{-q},$$

where $\epsilon_{\mathrm{matrix}}^{\prime }$ is the dielectric constant of the insulator matrix, *f*_c_ is the PT, *f* is the volume fraction of conductive filler, and *q* is a critical component. As shown in Figure 3, the dependence of ϵ′ on the volume fraction of Au NPs can be well described by Eq. (1). From the fitted result, *f*_*c*_ and *q* were found to be 0.21 and 0.55, respectively. In the case where conductive fillers were in spherical form, the PT of the two-phase random composite was theoretically calculated to be 0.16 [22,24]. *f*_c_ of the CCTO/Au system was larger than the calculated value (0.16). However, the critical exponent (*q* ≈ 0.55) was lower than the lower limit of the normal range (*q* ≈ 0.8 to 1), indicating a slow increase in ϵ′ with increasing metal content. Deviation of *f*_c_ and *q* from percolation theory may be due to the agglomeration of Au NPs to form large Au particles in the CCTO matrix, as clearly seen in Figure 2d. *f*_c_ of the CCTO/Au system is comparable to those observed in the Ba_0.75_Sr_0.25_TiO_3_/Ag (*f*_c_ = 0.285) [9] and BaTiO_3_/Ni (*f*_c_ = 0.232 to 0.310) [4,7] microcomposite systems. In the cases of the nanocomposite systems of PbTiO_3_/Ag [8] and Pb_0.4_Sr_0.6_TiO_3_/Ag [11], *f*_c_ values were found to be 0.16. Actually, the obtained *f*_c_ and *q* might not be highly accurate values or not the best values due to a large range of Au NPs volume fraction between 0.1 and 0.2. However, one of the most important factors for the observed higher *f*_c_ for the CCTO/Au system clearly suggested a morphology transition from nanocomposite to microcomposite as Au NP concentration was increased to 20 vol.%. This result is consistent to the microcomposite systems of Ba_0.75_Sr_0.25_TiO_3_/Ag [9] and BaTiO_3_/Ni [4,7]. Generally, the distribution of fillers in a matrix has an influence on the value of *f*_c_. For spherical fillers, *f*_c_ of randomly distributed fillers is given by the ratio between the particle size of the matrix phase (*R*_1_) and the filler (*R*_2_) [22]. When *R*_1_/*R*_2_ ≈ 1 or *R*_1_ ≈ *R*_2_, we obtain *f*_*c*_ ≈ 0.16. As *R*_1_/*R*_2_ > > 1 or *R*_1_ > > *R*_2_, the fillers fill the interstitial space between the matrix phase particles, resulting in a continuous percolating cluster of the filler at *f*_c_ *<* 0.16. As shown in Figure 2, the particle size of CCTO (*R*_1_) is larger than that of Au NPs (*R*_2_), i.e., *R*_1_/*R*_2_ > > 1. Theoretically, *f*_c_ of the CCTO/Au NP system should be lower than 0.16. However, the observed *f*_c_ value in the CCTO/Au system was found to be 0.21. Therefore, it is strongly indicated that the primary factor that has a great effect on *f*_c_ is the agglomeration of the Au filler.

Large increases in ϵ′ of percolating composites are generally attributed to formation of microcapacitor networks in the composites and/or Maxwell-Wagner polarization [4,9,22]. For pure CCTO ceramics, the giant dielectric response is normally associated with the mean grain size [16,17,25]. Although there is a small amount of relatively large grains (5 to 10 μm) in the microstructure of CCTO/Au3 and CCTO/Au4 (data not presented), the large observed enhancement of ϵ′ is likely due to the percolation effect.

**Figure 4:**
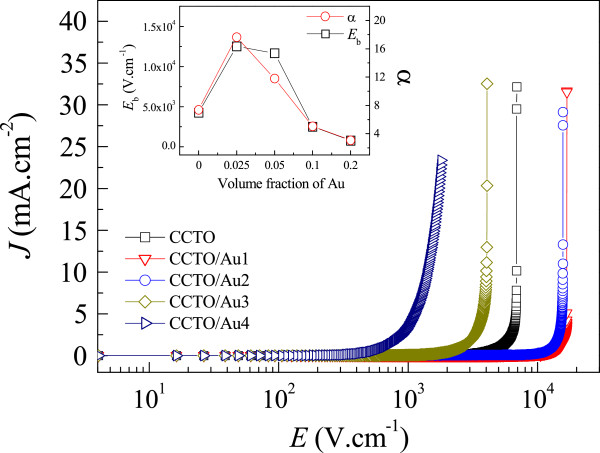
***J*-*E *curves of CCTO/Au nanocomposites.** The inset shows values of *E*_b_ and α as a function of Au concentration.

According to the effective medium theory [26], the average microscopic electric field inside the ceramic matrix filled with conductive particles increases in the region of the PT, which results in a significant decrease in *E*_b_. Figure 4 shows the non-Ohmic properties of the CCTO/Au nanocomposites as a plot of electrical current density (*J*) vs. electric field strength (*E*). α values of the CCTO, CCTO/Au1, CCTO/Au2, CCTO/Au3, and CCTO/Au4 samples were calculated in the range of *J* = 1 to 10 mA/cm^2^ and found to be 7.38, 17.67, 11.08, 5.05, and 3.08, respectively. *E*_b_ values (obtained at *J* = 1 mA/cm^2^) were found to be 4.26 × 10^3^, 1.25 × 10^4^, 1.17 × 10^4^, 2.50 × 10^3^, and 7.84 × 10^2^ V/cm, respectively. α and *E*_b_ initially showed a strong increase with introduction of 2.5 to 5.0 vol.% of Au NPs into CCTO (inset of Figure 4). Both parameters greatly decreased with further increasing Au NPs from 10 to 20 vol.%, which is due to the percolation effect [4]. In the region of the PT, electrical conduction in composites increased dramatically, resulting in a large decrease in *E*_b_. This observation is consistent with the effective medium theory [26]. Therefore, it is reasonable to suggest that the increases in ϵ′ and tanδ observed in the CCTO/Au4 sample were mainly attributed to the percolation effect; while, the effect of grain size effect is slight.

The CCTO/Au1 sample exhibited the best non-Ohmic properties among all samples. These values are comparable to those observed in CaCu_3_Ti_3.8_Sn_0.2_O_12_ ceramic [27]. There are many factors that are potentially responsible for strong improvement of non-Ohmic properties. It was found that the non-Ohmic properties of CCTO ceramics could effectively be improved by fabricating composite systems of CCTO/CTO [28,29]. As shown in Figure 1, the observed CTO phase in all of the CCTO/Au composites tended to increase with increasing Au content. However, the non-Ohmic properties of CCTO/Au strongly degraded as the Au filler concentration increased. Thus, the excellent non-Ohmic properties of the CCTO/Au1 sample are not mainly caused by a CTO phase. For CCTO polycrystalline ceramics, the non-Ohmic behavior is due to the existence of Schottky barriers at the GBs [13]. Thus, the existence of metallic Au NPs at the GBs of CCTO ceramics may contribute the formation of Schottky barriers at GBs. However, the mechanism by which Au NPs contribute to enhancement of non-Ohmic properties is still unclear.

It is worth noting that improved nonlinear properties of the CCTO/Au1 sample may also be related to modification of microstructure. Although the introduction of metallic particles in a ceramic matrix with concentration near the PT can dramatically enhance the dielectric response, a large increase in the conduction of charge carriers was observed simultaneously, leading to decreases in *E*_b_ and energy density. The maximum stored energy densities of all the samples were calculated and found to be 3.11, 25.8, 26.0, 1.39, and 0.54 kJ/m^3^ for the CCTO, CCTO/Au1, CCTO/Au2, CCTO/Au3, and CCTO/Au4 samples, respectively. Notably, introduction of Au NPs into CCTO ceramics in small concentrations, between 2.5 and 5.0 vol.%, caused a strong increase in the maximum stored energy density as well as their non-Ohmic properties.

## Conclusions

In conclusion, the investigation of non-Ohmic and dielectric properties of CCTO/Au revealed that addition of Au NPs to CCTO in the concentration of 2.5 vol.% can decrease tanδ, while ϵ′ was unaltered. The non-Ohmic properties of this composition were also successfully improved showing α ≈ 17.7 and *E*_b_ ≈ 1.25 × 10^4^ V/cm. The maximum stored energy density of CCTO ceramics were significantly enhanced by introducing of Au NPs in concentrations of 2.5 to 5.0 vol.%. The dielectric and non-Ohmic properties as well as energy density were degraded when Au NP concentrations were greater. The mechanisms of dielectric response and non-Ohmic properties can be well described by using the percolation theory.

## Competing interests

The authors declare that they have no competing interests.

## Authors’ contributions

WT carried out all the experiments, except for the preparation of Au nanoparticles. SS prepared Au nanoparticles. BP and TY offered technical support for the dielectric and I-V measurements. AC and PT supervised the research, designed the experiments, and participated in preparing the draft of the manuscript. PT revised the manuscript. VA and SM gave suggestions on the study. All authors read and approved the final manuscript.
